# Supplementary material for: Identification of photoperiod-induced specific miRNAs in the adrenal glands of Sunite sheep (Ovis aries)
Source: Front Vet Sci. 2022 Jul 22;9:888207. doi: 10.3389/fvets.2022.888207 (PMC9354845; doi:10.3389/fvets.2022.888207)
Supplement: Supplementary file 1 [file Data_Sheet_1.ZIP › Supplementary file/miRDeep2 file.zip!/miRDeep2 file/Know_Structure/LP42A1/oar-mir-376b.pdf]

miRBase precursor	: oar-mir-376b
Total read count	: 563
oar-miR-376e-5p read count	: 221
oar-miR-376b-5p read count	: 221
oar-miR-376b-3p read count	: 121
remaining reads	: 0

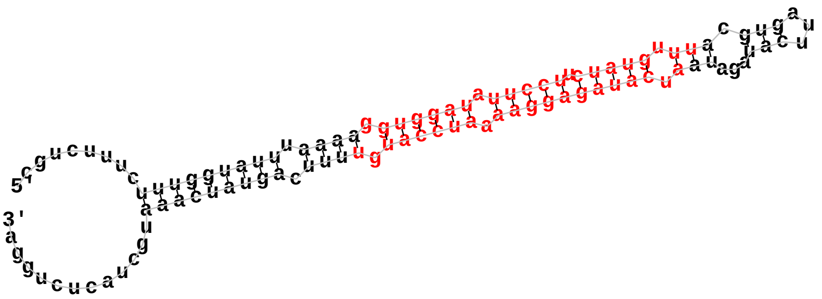

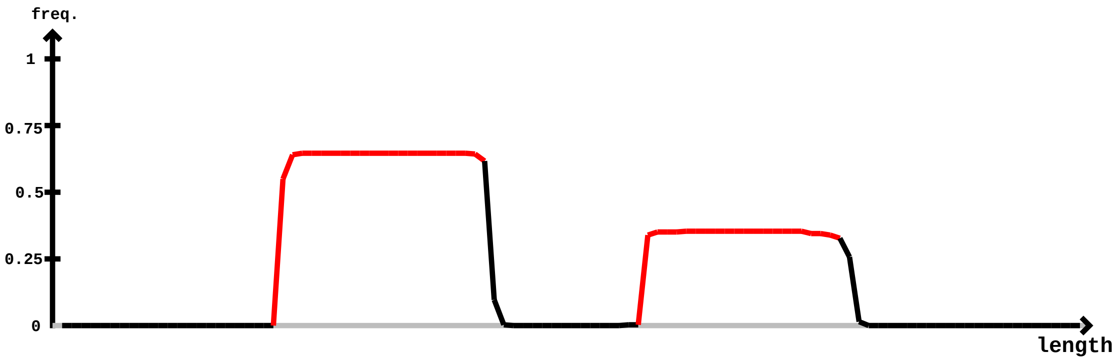


```
                                                             oar-miR-376b-3p
                       oar-miR-376b-5p
                       oar-miR-376e-5p
5'- cgucuuucuuugguauuuaaaagguggauauuccuucuauguuuacgugauucauagauaaucauagaggaaaauccauguuuucaguaucaaaugcuacucugga   -3'   exp
    ........(((((((((.((((.((((((.(((((.(((((.(((.(((...)))...))).)))))))))).)))))).)))).))))))))).............   reads   mm   sample
    ......................gguggauauuccuucuaugu................................................................   1       0    seq
    ......................gguggauauuccuucuauguu...............................................................   7       0    seq
    ......................gguggauauuccuucuauguuu..............................................................   154     0    seq
    ......................gguggauauuccuucuauguuua.............................................................   25      0    seq
    ......................gguggauauuccuucuauguuuac............................................................   1       0    seq
    .......................guggauauuccuucuauguu...............................................................   2       0    seq
    .......................guggauauuccuucuauguuu..............................................................   23      0    seq
    .......................guggauauuccuucuauguuua.............................................................   6       0    seq
    ........................uggauauuccuucuauguuu..............................................................   1       0    seq
    ........................uggauauuccuucuauguuua.............................................................   1       0    seq
    ..........................................................uaaucauagaggaaaauccaug..........................   1       0    seq
    ............................................................aucCuagaggaaaaucc.............................   1       1    seq
    ............................................................aucauagaggaaaaucc.............................   2       0    seq
    ............................................................aucauagaggaaaauccau...........................   2       0    seq
    ............................................................aucauagaggaaaauccaug..........................   3       0    seq
    ............................................................aucauagaggaaaauccaugu.........................   24      0    seq
    ............................................................aucauagaggaaaauccauguu........................   81      0    seq
    ............................................................aucauagaggaaaauccauguuu.......................   2       0    seq
    .............................................................ucauagaggaaaauccauguu........................   1       0    seq
    .............................................................ucauagaggaaaauccauguuu.......................   3       0    seq
    ................................................................uagaggaaaauccauguu........................   1       0    seq
```
